# MicroRNAs in immune-related diseases: mechanism, functions and therapeutic perspectives

**DOI:** 10.3389/fimmu.2026.1943071

**Published:** 2026-09-04

**Authors:** Tutku Tunç, Serap Yaman Ozer, Sema Misir

**Affiliations:** 1 Department of Pharmaceutical Microbiology, Faculty of Pharmacy, Sivas Cumhuriyet University, Sivas, Türkiye; 2 Department of Medical Biochemistry, Faculty of Medicine, Trabzon Kanuni Training and Research Hospital, Trabzon University, Trabzon, Türkiye; 3 Department of Biochemistry, Faculty of Pharmacy, Sivas Cumhuriyet University, Sivas, Türkiye

**Keywords:** atopic dermatitis (AD), biomarker, inflammatory bowel disease (IBD), microRNAs(miRNAs), multiple sclerosis (MS), psoriasis, rheumatoid arthritis (RA), systemic lupus erythematosus (SLE)

## Abstract

MicroRNAs (miRNAs) are evolutionarily conserved, non-coding RNA molecules approximately 18–25 nucleotides in length that regulate gene expression at the post-transcriptional level. Their principal mechanism of action is post-transcriptional gene silencing via RNA interference, achieved by binding to complementary sequences within target messenger RNAs (mRNAs). Accumulating evidence has highlighted the pivotal role of miRNAs in the development, differentiation, and function of immune cells, as well as in maintaining immune homeostasis. A more comprehensive understanding of the complex molecular networks governed by these miRNAs may provide valuable insights into disease mechanisms, facilitate clinical decision-making, and ultimately improve patient outcomes. The remarkable stability of miRNAs, together with their presence in the systemic circulation encapsulated within extracellular vesicles, has attracted considerable interest in their clinical application. These characteristics make circulating miRNAs promising candidates as diagnostic biomarkers for the early detection of disease and as prognostic indicators for disease progression and evaluating therapeutic efficacy. Furthermore, an increasing number of miRNA-based studies across diverse immune-related disorders have revealed their potential as therapeutic targets. The integration of synergistic therapeutic strategies and complementary miRNA-based approaches may further enhance treatment efficacy and contribute to the development of novel precision-medicine interventions for immune-related diseases. In this review, we comprehensively discuss the biogenesis and biological functions of miRNAs and examine their regulatory roles in the pathogenesis of rheumatoid arthritis (RA), inflammatory bowel disease (IBD), multiple sclerosis (MS), psoriasis, systemic lupus erythematosus (SLE), and atopic dermatitis (AD). We are also evaluating the potential of these miRNAs as diagnostic and prognostic biomarkers, and their promising properties as therapeutic targets in the treatment of immune system-related diseases. These selected diseases represent key immune disorders in which miRNAs serve as strong biomarker candidates for early diagnosis (diagnostic) and disease course (prognostic) due to their stability in systemic circulation and their presence within extracellular vesicles. These six diseases constitute the cluster of “immune-related disorders,” where miRNA-based studies are steadily increasing, and these molecules hold the highest potential as novel therapeutic targets.

## 1 Introduction

The immune system is a highly sophisticated and tightly regulated biological network that protects the host against invading pathogens while preserving self-tolerance and tissue homeostasis. This intricate balance relies on coordinated interactions among innate and adaptive immune cells, soluble mediators, signaling molecules, and transcriptional regulators. Under physiological conditions, immune activation is rapidly initiated upon pathogen recognition and subsequently terminated through multiple regulatory mechanisms to prevent excessive inflammation and autoimmune tissue damage (1). Dysregulation of these finely orchestrated processes contributes to the initiation and progression of a wide spectrum of immune-mediated disorders, including autoimmune diseases, chronic inflammatory conditions, allergic diseases, infectious diseases, and cancer. Consequently, elucidating the molecular mechanisms governing immune homeostasis has become one of the major objectives of modern immunology (2, 3). Although autoimmune, autoinflammatory, allergic, and chronic inflammatory diseases differ considerably in their clinical manifestations and affected organs; they share several common molecular mechanisms, including impaired immune tolerance, persistent activation of innate and adaptive immune responses, dysregulated cytokine production, and chronic inflammation. Increasing evidence indicates that these shared mechanisms are coordinated by complex post-transcriptional regulatory networks, among which microRNAs have emerged as central modulators of immune homeostasis. Among the regulatory mechanisms controlling immune responses, epigenetic regulation has emerged as a fundamental determinant of immune cell development and function. Unlike genetic alterations, epigenetic modifications dynamically regulate gene expression without changing the underlying DNA sequence, allowing immune cells to rapidly adapt to environmental cues and inflammatory stimuli (4). DNA methylation, histone modifications, chromatin remodeling, and non-coding RNAs collectively constitute the major epigenetic mechanisms that control immune cell differentiation, activation, and maintenance of immune tolerance. Increasing evidence suggests that disruption of these epigenetic regulatory networks contributes directly to chronic inflammation, autoimmunity, and immune dysfunction, highlighting their importance in both physiological and pathological immune responses (4, 5). Among epigenetic regulators, microRNAs (miRNAs) have attracted considerable attention for their ability to simultaneously regulate multiple genes involved in immune signaling pathways. miRNAs are evolutionarily conserved endogenous non-coding RNAs approximately 18–25 nucleotides in length that negatively regulate gene expression at the post-transcriptional level (6). By binding predominantly to complementary sequences located within the 3′ untranslated region (3′UTR) of target messenger RNAs (mRNAs), mature miRNAs induce translational repression and/or mRNA degradation, thereby fine-tuning protein synthesis. Unlike classical transcription factors that generally regulate a limited number of downstream genes, a single miRNA can modulate hundreds of target transcripts.

**Figure 1:**
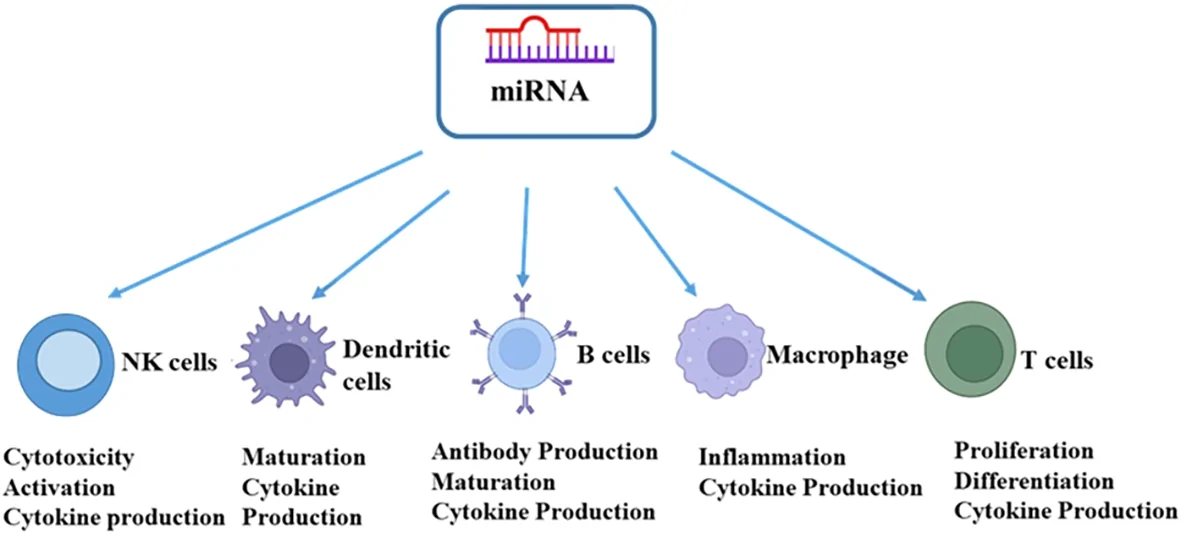
The role of miRNAs in immune regulation. Created with BioRender.com.

In contrast, individual mRNAs may be simultaneously regulated by multiple miRNAs. Consequently, miRNAs function as master regulators of complex gene regulatory networks rather than simple on/off molecular switches (6, 7). Compared with conventional protein biomarkers and individual cytokines, miRNAs possess several unique biological advantages, including remarkable molecular stability, tissue-specific expression patterns, evolutionary conservation, and the capacity to simultaneously regulate multiple signaling pathways. These characteristics make miRNAs particularly attractive candidates for both mechanistic studies and clinical applications. The immune system represents one of the biological systems most extensively controlled by miRNAs. Appropriate immune responses require precise temporal and spatial regulation of immune cell differentiation, lineage commitment, activation thresholds, cytokine production, and the resolution of inflammation (8). Many miRNAs have been identified as indispensable regulators of hematopoiesis and immune cell maturation, influencing the development and functional specialization of macrophages, dendritic cells, neutrophils, natural killer (NK) cells, B lymphocytes, CD4^+^ helper T cells, CD8^+^ cytotoxic T cells, and regulatory T cells (Tregs). Furthermore, miRNAs participate in antigen presentation, immune checkpoint regulation, inflammasome activation, pattern-recognition receptor signaling, and cytokine-mediated intercellular communication. These regulatory effects establish miRNAs as critical molecular checkpoints that prevent excessive immune activation while ensuring effective host defense (8, 9) (**Figure 1**).

Accumulating evidence demonstrates that aberrant miRNA expression is closely associated with immune-mediated diseases. Altered miRNA signatures have been identified in rheumatoid arthritis (RA), systemic lupus erythematosus (SLE), multiple sclerosis (MS), inflammatory bowel disease (IBD), psoriasis, atopic dermatitis (AD), systemic sclerosis, and many other chronic inflammatory disorders (10). Disease-associated miRNAs regulate multiple pathogenic mechanisms, including macrophage polarization, Th1/Th2/Th17 differentiation, Treg dysfunction, B-cell activation, autoantibody production, cytokine storms, oxidative stress, fibrosis, and endothelial dysfunction (10–12).

Although many reviews have discussed the role of miRNAs in individual autoimmune or inflammatory disorders, a comprehensive comparison integrating immune regulation, disease-specific mechanisms, biomarker development, and therapeutic applications across major immune-mediated diseases is limited. Therefore, this review aims to bridge these areas by providing an integrated perspective that connects fundamental miRNA biology with emerging clinical applications. In this review, we provide a comprehensive overview of the current understanding of miRNA biology with particular emphasis on their biogenesis, molecular mechanisms of action, and regulatory roles in both innate and adaptive immunity. We further summarize recent evidence regarding the involvement of miRNAs in major immune-related diseases, discuss their emerging utility as diagnostic and prognostic biomarkers, and highlight current advances in miRNA-based therapeutic strategies. Finally, we address the major challenges and future perspectives for translating miRNA research into clinical immunology and precision medicine.

## 2 General overview of miRNA biology

### 2.1 Historical perspective and biological significance of miRNAs

The discovery of miRNAs fundamentally transformed our understanding of gene regulation by revealing that protein-coding genes are not the sole determinants of cellular phenotype and biological complexity. For decades, the central dogma of molecular biology emphasized that genetic information flows from DNA to RNA and subsequently to proteins, suggesting that proteins were the principal functional molecules responsible for cellular activities. However, the identification of small non-coding regulatory RNAs demonstrated that RNA molecules themselves possess extensive regulatory functions independent of protein synthesis. This paradigm shift established non-coding RNAs as critical regulators of gene expression and initiated an entirely new era in molecular biology, developmental genetics, and systems immunology (13). The remarkable biological versatility of miRNAs has also generated considerable interest in their translational applications. Because miRNA expression changes dynamically during disease initiation and progression, disease-specific miRNA signatures have been identified in autoimmune disorders, infectious diseases, cardiovascular diseases, neurodegenerative disorders, metabolic diseases, and virtually every major cancer type. Furthermore, the exceptional stability of circulating miRNAs in serum, plasma, urine, saliva, and other body fluids has established these molecules as promising, minimally invasive biomarkers for diagnosis, prognosis, monitoring of therapeutic response, and precision medicine. Simultaneously, advances in RNA therapeutics have enabled the development of miRNA mimics and antisense oligonucleotides (antagomiRs), several of which have already entered clinical trials, highlighting the growing therapeutic potential of miRNA-based interventions (14–16).

### 2.2 miRNA biogenesis and functions

**Figure 2:**
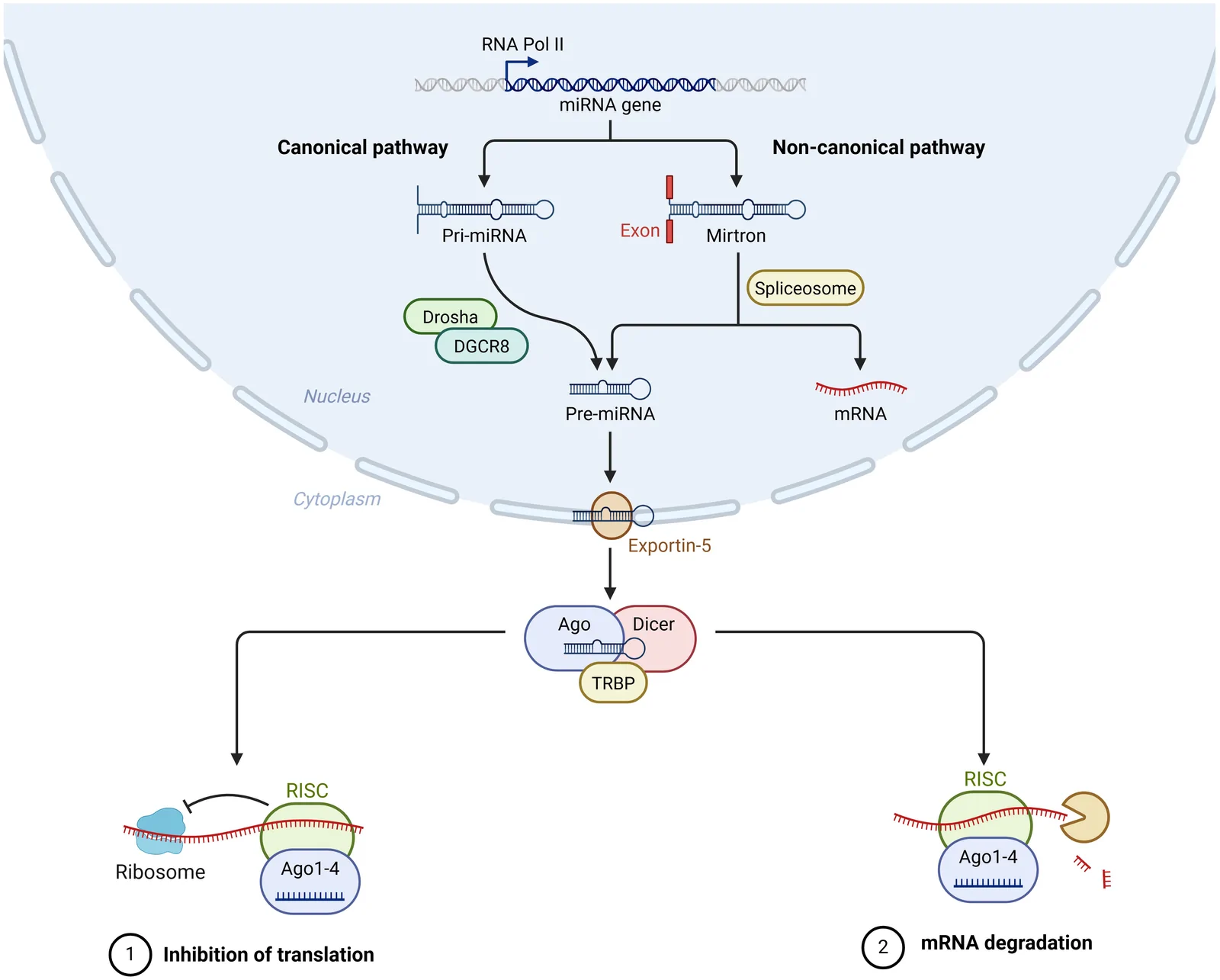
miRNA biogenesis and the miRNA-mRNA interaction mechanism. Primarily, mRNA degradation or decreased translational efficiency. Created with BioRender.com.

miRNAs are generated through a highly coordinated and evolutionarily conserved multistep maturation process that ensures precise temporal and spatial regulation of gene expression. Unlike messenger RNAs, which function as templates for protein synthesis, miRNAs exert their biological activities by guiding post-transcriptional repression of target transcripts. Consequently, the efficiency of miRNA biogenesis directly determines the magnitude and specificity of gene silencing, making every step of the maturation pathway subject to strict molecular regulation. Increasing evidence indicates that disturbances occurring during miRNA biogenesis contribute not only to abnormal gene expression but also to immune dysregulation, chronic inflammation, tumorigenesis, and numerous human diseases. Therefore, understanding the molecular mechanisms governing miRNA biogenesis provides the essential framework for interpreting both physiological and pathological functions of miRNAs (17). The canonical miRNA biogenesis pathway begins in the nucleus, where miRNA genes are primarily transcribed by RNA polymerase II (RNA Pol II). However, a subset of miRNAs is transcribed by RNA polymerase III, depending on their genomic organization. Similar to protein-coding genes, RNA Pol II-derived primary miRNA transcripts (pri-miRNAs) undergo co-transcriptional 5′ capping with 7-methylguanosine and 3′ polyadenylation, indicating that miRNA genes share many regulatory features with conventional transcriptional units. Pri-miRNAs vary considerably in length, ranging from several hundred nucleotides to more than ten kilobases, and may contain one or multiple imperfect stem-loop structures. These transcripts can originate from independent intergenic loci, introns of protein-coding genes, exons of non-coding genes, or polycistronic miRNA clusters, thereby allowing coordinated expression of multiple functionally related miRNAs from a single transcriptional unit. This genomic diversity contributes significantly to the complexity of miRNA-mediated regulatory networks (17, 18). Following transcription, pri-miRNAs undergo the first maturation step through the action of the Microprocessor complex, one of the most sophisticated RNA-processing machineries identified in eukaryotic cells. This nuclear complex consists principally of the RNase III endonuclease Drosha and its essential RNA-binding partner DGCR8 (DiGeorge syndrome critical region gene 8). Drosha serves as the catalytic component responsible for RNA cleavage, whereas DGCR8 functions as a molecular sensor that recognizes the characteristic stem-loop architecture of pri-miRNAs and accurately positions Drosha at the cleavage site. Structural studies have demonstrated that DGCR8 recognizes the junction between double-stranded stem regions and adjacent single-stranded RNA segments, thereby enabling highly precise processing of thousands of distinct pri-miRNA transcripts despite their considerable sequence variability (19). Drosha-mediated cleavage occurs approximately 11 base pairs from the basal junction of the hairpin structure, generating an approximately 60-70-nucleotide precursor miRNA (pre-miRNA) with the characteristic two-nucleotide 3′ overhang recognized by downstream transport machinery. This processing step is among the most tightly regulated events in the miRNA biogenesis pathway, as even minor alterations in Drosha cleavage accuracy can shift the mature miRNA sequence, particularly within its seed region (nucleotides 2–8), thereby profoundly altering target specificity (20). After nuclear processing, precursor miRNAs are actively transported to the cytoplasm through the Exportin-5/Ran-GTP transport system. Once the complex reaches the cytoplasm, hydrolysis of Ran-bound GTP induces conformational changes that release pre-miRNAs for subsequent maturation (19). Within the cytoplasm, pre-miRNAs undergo the second major processing step catalyzed by Dicer, another RNase III family endonuclease. Dicer cooperates with accessory proteins, particularly the transactivation response RNA-binding protein (TRBP) and protein activator of PKR (PACT), to precisely remove the terminal loop of the precursor hairpin. This cleavage generates an approximately 22-nucleotide miRNA duplex consisting of a guide strand and a complementary passenger strand. The interaction between Dicer and TRBP also facilitates the subsequent loading of the mature guide strand into the RNA-induced silencing complex (RISC), thereby coupling miRNA maturation directly to functional activation. Unlike the canonical miRNA biogenesis pathway, non-canonical pathways allow miRNAs to be produced via alternative cellular mechanisms without requiring either Drosha or Dicer. In these alternative processes, mirnads, rather than being cleaved by Drosha in the nucleus, are directly converted into pre-miRNA-like structures via mRNA splicing, thereby participating in biogenesis. In another important mechanism, the Dicer-independent pathway, precursor molecules transported to the cytoplasm are not cleaved by Dicer but are directly processed by the Argonaute 2 (Ago2) protein to mature into miRNAs. These non-canonical pathways enable immune cells to mount more flexible and rapid molecular responses to environmental stimuli and pathological signals, thereby expanding the regulatory capacity of miRNAs in immune homeostasis and disease pathogenesis (19, 20) (**Figure 2**).

Importantly, several regulatory checkpoints influence miRNA maturation. Mutations or altered expression of Drosha, DGCR8, Dicer, Exportin-5, or Argonaute proteins have been associated with immune dysregulation, chronic inflammation, and autoimmune diseases, emphasizing that abnormalities in miRNA biogenesis may contribute directly to disease pathogenesis rather than simply altering miRNA abundance.

## 3 miRNAs in immune-related diseases

miRNAs have been shown to exhibit highly specific expression patterns in immune-associated organs, suggesting they play roles in the development, maturation, activation, proliferation, function, and survival of various immune cells. It is well known that dysregulated miRNA expression can lead to impaired immune tolerance and the development of immune-related diseases. With the increasing recognition that miRNAs play critical roles in regulating immune responses and immune cell development, current research has focused on the relationship between miRNAs and immune-related diseases (21). As shown in **Table 1**, miRNAs play a regulatory role in the development of immune-related diseases. For example, the simultaneous increase in molecules such as miR-155 and miR-223 across multiple diseases suggests that they are central regulators of general immune activation and inflammation rather than of specific diseases. Therefore, for miRNA-based findings to become reliable diagnostic tools, it is necessary to identify disease-specific “miRNA signatures” rather than individual molecules and to integrate these data with clinical findings in line with precision medicine approaches.

**Table 1:** miRNAs play a regulatory role in the development of immune-related diseases.

miRNA	Expression	Target(s)	Disease	Ref
miR-155	Up	MMP-3	RA	(22)
miR -146a	Up	IRAK1, TRAF6, FAF1	RA	(23, 24)
miR-346	Up	BTK, TNF-α	RA	(25, 26)
miR-124a	Down	CDK-2, MCP-1	RA	(27)
miR-34a	Down	XIAP	RA	(28)
miR-143-3p	Up	IGF1R, IGFBP5	RA	(29)
miR-338-5p	Up	NFAT5	RA	(30)
miR-192	Up	CAV1	RA	(31)
miR-146a	Up	IL-17, TRAF6, IRAK1	PS	(32, 33)
miR-203	Up	TP63	PS	(34)
miR-203	Up	LXR-α, PPAR-γ	PS	(35)
miR-203	Up	SOCS3, SOCS6, TP63, TNF-α, IL-8, IL-24	PS	(36)
miR-21	Up	MEG3	PS	(37)
miR-31	Up	PPP6C, ET-1, STK40, TNF-α, IL-1, IL-6, IL-17, IL-22	PS	(38–40)
miR-99a	Down	FZD5/FZD8	PS	(41)
miR-125b	Down	BRD4	PS	(42)
miR-155	Down	PTEN	PS	(43)
miR-96	Up		MS	(44)
miR-326	Up	ETS-1	MS	(45)
miR-21, miR-155, miR-182	Up		MS	(46)
miR-467b	Up	EIF4E	MS	(47)
miR-155, miR-326, miR-34a	Up		MS	(48)
miR-92	Up		MS	(49)
miR-17-5p	Up	PTEN	MS	(50)
miR-21, miR-148a	Up	DNMT1	SLE	(51)
miR-21	Up	PDCD4	SLE	(52)
miR-29b	Up	DNMT1	SLE	(53)
miR-126	Up	DNMT1	SLE	(54)
miR-181a	Down	PCAF	SLE	(55)
miR-146a	Down	TRAF6	SLE	(56)
miR-125a	Down	KLF13	SLE	(57)
miR-155	Up	CD62L	SLE	(58)
miR-183C	Up	FOXO1	SLE	(59)
miR-196a	Down	HOX-C8	SLE	(60)
miR-1246	Up	EBF1	SLE	(61)
miR-152-3p	Up	KLF5	SLE	(62)
miR-192	Down Up	MIP-2 NOD2	IBD	(63, 64)
miR-21	Up	PDCD4, RHOB	IBD	(65, 66)
miR-31, miR-155	Up	IL13Rα1	IBD	(67)
miR-29	Up	IL-12P40	IBD	(68)
miR-122	Up	Occludin	IBD	(69)
miR-141	Down	CXCL5, CXCL12	IBD	(70, 71)
miR-150	Up	C-MYB	IBD	(72)
MiR-124	Down	IFN-γ, TNF- α	AD	(73)
miR-143		IL-13Rα1	AD	(74)
miR-151a	Up	IL12RB2	AD	(75)
miR-155	Up	CTLA-4	AD	(76)
miR-335	Down	SOX6	AD	(77)

RA, rheumatoid arthritis; PS, psoriasis; MS, multiple sclerosis; SLE, systemic lupus erythematosus; IBD, Inflammatory bowel disease; AD, atopic dermatitis; MMP-3,matrix metalloproteinase-3; IRAK1, interleukin-1 receptor-associated kinase 1 TRAF6, Tumor Necrosis Factor (TNF) Receptor-Associated Factor 6; FAF1, Fas-associated factor 1; *BTK*, Bruton tyrosine kinase; TNF-α,Tumor Necrosis Factor alpha; CDK-2, Cyclin-dependent kinase 2; MCP-1, Monocyte Chemoattractant Protein-1; XIAP, X-linked inhibitor of apoptosis protein; IGF1R, Insulin-like Growth Factor 1 Receptor; IGFBP5, Insulin-like growth factor-binding protein 5; NFAT5, Nuclear factor of activated T-cells 5; CAV1,Caveolin 1; IL-17, Interleukin-17; P63, Tumor Protein 63; LXR-α, Liver X Receptor alpha; PPAR-γ, eroxisome proliferator-activated receptor gamma; SOCS3, Suppressor of Cytokine Signaling 3; SOCS6, Suppressor of Cytokine Signaling 6; IL-8, Interleukin-8; IL-24, Interleukin-24; MEG3, Maternally Expressed Gene 3; PPP6C, protein phosphatase 6; ET-1,endothelin-1; STK40, erine/threonine-protein kinase 40; FZD5, Frizzled-5; FZD8, Frizzled-5; PDCD4, programmed cell death protein 4; DNMT1, DNA methyltransferase 1; KLF13, Kruppel-like factor 13; BRD4, Bromodomain-containing protein 4; FOXO1, forkhead box protein O1; PTEN, Phosphatase and TENsin homolog; HOX-C8, homeobox transcription factor Hox-C8; EBF1, early B-cell factor 1; KLF5, Kruppel-like factor 5; MIP-2, macrophage inflammatory peptide-2; NOD2, nucleotide-binding oligomerization domain-containing protein 2; PDCD4, programmed cell death protein 4; RhoB, Ras homolog family member B; IL13R α1, interleukin-13 receptor alpha 1; CXCL5, C-X-C motif chemokine ligand 5; IL12RB2, interleukin-12 receptor beta 2; CTLA-4, cytotoxic T-lymphocyte-associated protein 4; SOX6, SRY-box transcription factor 6.

### 3.1 Rheumatoid arthritis

Rheumatoid arthritis is a chronic autoimmune disease characterized by persistent synovial inflammation that leads to tissue damage, affecting 0.2-1% of the world’s population (78). While the precise etiology of RA remains unclear, the dominant hypothesis is a genetic-environmental interaction in which the human leukocyte antigen (HLA)-DRB1 gene plays a significant role, and environmental triggers such as smoking, infection, and sex hormones act as risk factors for RA onset (79). RA pathogenesis can be defined in successive stages: synovial villus formation, inflammation, immune abnormalities, and cartilage-bone destruction (80). Treatment of RA can be challenging and often requires lifelong management, but advances in management paradigms and the development of more effective treatments have led to significant progress (81). Recent studies have highlighted the essential role of miRNAs in regulating immune responses and maintaining immune homeostasis, thereby preventing autoimmune disorders. In patients with RA, significant alterations in the expression profiles of various cellular miRNAs have been observed, suggesting that these molecules are actively involved in the pathogenesis and progression of the disease (82, 83). Altered expression profiles of several cellular miRNAs have been identified in patients with RA, particularly in peripheral blood mononuclear cells (PBMCs), T lymphocytes, synovial fibroblasts, and osteoclasts (83). These miRNAs participate in multiple signaling pathways involved in RA pathogenesis, including the nuclear factor of kappa light chain enhancer (NF-κB), phosphoinositide 3-kinase/protein kinase B (PI3K/Akt) signaling, apoptosis, and cytokine signaling. Aberrant miRNA expression influences cellular proliferation, apoptosis, and inflammatory responses, thereby contributing to disease progression (84). Among the dysregulated miRNAs, miR-155, miR-146, and miR-146a have consistently been reported to be overexpressed in synovial fibroblasts and synovial tissues from patients with RA (22, 85). miR-155 expression has been shown to suppress matrix metalloproteinase-3 (MMP-3) production in rheumatoid arthritis synovial fibroblasts (RASFs) and to counteract the induction of MMP-1 and MMP-3 mediated by pro-inflammatory cytokines and Toll-like receptor (TLR) ligands (22). In addition, many target genes of miR-155 have been identified, including *c-Maf*, *Bach1*, *PU.1*, *C/EBP*, and *SHIP1 (*86). McCoy et al. demonstrated that the anti-inflammatory cytokine interleukin-10 (IL-10) inhibits lipopolysaccharide (LPS)-induced transcription of miR-155 from the *BIC* gene in bone marrow-derived macrophages through a STAT3-dependent mechanism. This suppression subsequently increases SHIP1 expression, a direct target of miR-155 (87). miR-146a has also been implicated in RA pathogenesis by targeting interleukin-1 receptor-associated kinase 1 (IRAK1) and tumor necrosis factor receptor-associated factor 6 (TRAF6), two essential mediators of inflammatory signaling (23). Furthermore, miR-146a expression in CD4^+^ T cells isolated from synovial fluid has been reported to correlate positively with tumor necrosis factor-alpha (TNF-α) levels and negatively with Fas-associated factor 1 (FAF1), suggesting its involvement in regulating T-cell apoptosis (24). Another study demonstrated that miR-10a expression is significantly reduced in fibroblast-like synoviocytes (FLSs) derived from RA patients. Decreased miR-10a may promote NF-κB activation by targeting interleukin-1 receptor-associated kinase 4 (IRAK-4), transforming growth factor-β-activated kinase 1 (TAK1), and β-transducin repeat-containing protein (BTRC) (88). miR-346 has been shown to suppress the interleukin-18 (IL-18)-induced inflammatory response in fibroblast-like synoviocytes by inhibiting Bruton tyrosine kinase (BTK) transcription (25). In addition, miR-346 regulates the release of TNF-α, a pivotal pro-inflammatory cytokine in RA, by stabilizing tristetraprolin (TTP) (26). Reduced expression of miR-124a has also been associated with enhanced proliferation of RA synovial cells and dysregulated inflammatory responses. Cyclin-dependent kinase 2 (CDK2) and chemokine (C-C motif) ligand 2/monocyte chemoattractant protein-1 (CCL2/MCP-1) have been identified as direct targets of miR-124a, highlighting its importance in controlling synovial cell proliferation and inflammation (27). In synovial fibroblasts isolated from RA patients, basal expression levels of miR-34a have been reported to be significantly lower than those observed in osteoarthritis patients, whereas no significant differences have been detected for miR-34b or miR-34c expression. Restoration of miR-34a expression has been shown to enhance Fas ligand (FasL)- and TNF-related apoptosis-inducing ligand (TRAIL)-mediated apoptosis in rheumatoid arthritis synovial fibroblasts (28). In addition, miR-143-3p (29), miR-338-5p (30), and miR-192 (31) have been identified as important regulators of apoptosis and cell-cycle progression in RA. Although the precise molecular mechanisms underlying RA pathogenesis remain incompletely understood, continued investigation of miRNA-mediated regulatory networks is expected to provide valuable insights into the complex genetic and epigenetic mechanisms that drive disease initiation and progression, potentially facilitating the development of novel diagnostic biomarkers and targeted therapeutic strategies. Collectively, these findings suggest that RA-associated miRNAs regulate multiple interconnected signaling pathways rather than acting independently. Nevertheless, inconsistencies among studies regarding miRNA expression profiles may arise from differences in disease stage, treatment status, biological samples, and analytical platforms. Future multicenter studies using standardized methodologies are required before these miRNAs can be translated into clinical practice.

### 3.2 Psoriasis

Psoriasis is a common, immune-mediated, non-contagious, chronic inflammatory skin disease characterized by erythematous and scaly epidermal lesions. Its causes and treatment are still not fully understood. This lifelong condition affects individuals of all ages and occurs worldwide; reported prevalence rates range from 0.09% to 11.43% (115, 116). Psoriasis is characterized by excessive proliferation and abnormal differentiation of epidermal keratinocytes, along with infiltration of immune cells, including neutrophils, mast cells, innate lymphoid cells, and T cells. Elevated levels of inflammatory mediators, including TNF-α, interleukin (IL)-1, interferon (IFN)-γ, IL-17, and IL-22, contribute to the development of inflammation in psoriasis (115). miRNAs play essential regulatory roles in key cellular processes, including proliferation, apoptosis, and inflammation, all of which are central to disease pathogenesis. Many studies have shown that miRNA expression profiles in patients with psoriasis differ significantly from those in healthy individuals (117). Among the most extensively studied miRNAs, miR-146a is upregulated in psoriatic lesions and PBMCs, and in chronic inflammatory responses. It contributes to the suppression of innate immune activation in keratinocytes (118, 119). Reduced miR-146a expression has been associated with early disease onset, exacerbation of cutaneous inflammation, excessive IL-17 expression, and keratinocyte hyperproliferation (32, 119). miR-146a regulates TRAF6 and IRAK1, thereby attenuating NF-κB-dependent inflammatory signaling and modulating immune responses (33). miR-203, a keratinocyte-derived miRNA, is markedly upregulated in psoriasis and plays a critical role in disease pathogenesis (120). By targeting multiple epidermal genes, miR-203 regulates the balance between keratinocyte proliferation and differentiation (121). Its overexpression is associated with downregulation of suppressor of cytokine signaling 3 (SOCS3) and subsequent activation of signal transducer and activator of transcription 3 (STAT3), which enhances cytokine–keratinocyte interactions. For this reason, miR-203 has been proposed as a potential therapeutic target in psoriasis (117). In addition, miR-203 binds to the 3′UTR of p63 mRNA, inducing cell cycle arrest at the G0/G1 phase and promoting keratinocyte differentiation (34). It also negatively regulates liver X receptor alpha (LXR-α) and peroxisome proliferator-activated receptor gamma (PPAR-γ), suggesting that the miR-203–LXR-α/PPAR-γ axis contributes to the hyperproliferative phenotype of psoriatic keratinocytes and may represent a potential therapeutic pathway (35). Furthermore, miR-203 suppresses the expression of pro-inflammatory mediators, including TNF-α, IL-8, and IL-24, by directly targeting their mRNAs. Consistent with these findings, Mostafa et al. reported significant upregulation of miR-203 in psoriasis, accompanied by downregulation of its target genes, including SOCS3, SOCS6, TP63, TNF-α, IL-8, and IL-24 (36). miR-21 is another key regulator that is significantly upregulated in psoriasis and is involved in multiple cellular processes, including proliferation, differentiation, apoptosis, and migration, through its specific target genes (38). It is expressed in psoriatic skin lesions, epidermal keratinocytes, dermal T cells, and blood samples (39). miR-21 regulates keratinocyte proliferation and apoptosis by binding to the 3′UTR of caspase-8 mRNA (37). Within keratinocytes (122), it promotes cell survival, proliferation, and resistance to apoptosis by inhibiting negative regulators such as phosphatase and tensin homolog (PTEN) and programmed cell death protein 4 (PDCD4), consistent with early observations in psoriatic skin. miR-21 expression also enhances intercellular communication between the immune and epidermal compartments, thereby contributing to disease persistence (123). Many studies have demonstrated that miR-31 targets protein phosphatase 6 catalytic subunit (PPP6C), factor inhibiting hypoxia-inducible factor 1 (FIH-1), endothelin-1 (ET-1), and STK40, indicating its involvement in psoriasis pathogenesis (38, 39). Moreover, miR-31 is implicated in the regulation of key signaling pathways, including NF-κB, Notch, and RAS/MAPK. It modulates the production of inflammatory mediators, including TNF-α, IL-1, IL-6, IL-17, and IL-22, and promotes leukocyte chemotaxis (40). Therefore, inhibition of miR-31 has been proposed as a potential therapeutic strategy in psoriasis, given its central role in keratinocyte regulation and inflammatory signaling (39). Expression of miR-99a is reduced in PBMCs and skin lesions from patients with psoriasis (41, 124). This miRNA targets insulin-like growth factor 1 receptor (IGF-1R), which contributes to basal keratinocyte proliferation, epidermal hyperplasia, and hyperkeratosis (124). miR-99a suppresses IGF-1R protein expression, thereby inhibiting keratinocyte proliferation and promoting terminal differentiation. In addition to IGF-1R, frizzled receptors FZD5 and FZD8 have also been validated as direct targets of miR-99a. Shen et al. reported that miR-99a inhibits keratinocyte proliferation by suppressing the FZD5/FZD8 axis (41). miR-125b is one of the most downregulated miRNAs in psoriatic keratinocytes (42). It plays a role in psoriasis pathogenesis by targeting fibroblast growth factor receptor 2 (FGFR2), AKT3, and ubiquitin-specific peptidase 2 (USP2) (121). Under physiological conditions, miR-125b contributes to the regulation of epidermal renewal by targeting FGF7, MAPK14, and BLIMP1, thereby restricting cellular proliferation. Loss of miR-125b disrupts keratinocyte differentiation programs and contributes to epidermal hyperplasia independently of immune cell infiltration (42). miR-155 is significantly upregulated in psoriatic skin lesions and PBMCs, where it plays crucial roles in keratinocyte proliferation, apoptosis, and inflammatory responses (121). It regulates the differentiation of CD4^+^ T cells into Th1 and Th2 subsets and is further induced upon T-cell activation. miR-155 also promotes differentiation of Treg and Th17 cells. Additionally, it targets PTEN, a negative regulator of PI3K/AKT signaling, thereby contributing to enhanced inflammatory signaling (43). miR-197 is downregulated in psoriatic skin and is involved in reducing keratinocyte proliferation and migration while promoting normal differentiation processes (39). It has also been associated with modulation of IL-22 and IL-17 receptor signaling pathways, influencing keratinocyte proliferation and differentiation dynamics (125). These findings highlight the central role of miRNAs in regulating inflammatory and proliferative pathways in psoriasis and underscore their potential as therapeutic targets.

### 3.3 Multiple sclerosis

Multiple sclerosis is an autoimmune disorder affecting the central nervous system (CNS), characterized by demyelination, axonal damage, axonal fragmentation, and the formation of numerous localized sclerotic lesions (126). This manifests itself in motor, sensory, and therapeutic ways. The fundamental mechanisms in the onset and course of MS are not yet fully understood. Recent studies on the pathophysiology of MS suggest that inflammation is triggered by T cell proliferation, leading to the release of pro-inflammatory cytokines in axons, changes in the permeability of the blood-cerebrospinal fluid barrier, axonal swelling, macrophage activation, the development of a demyelination reaction, and pro-inflammatory miRNAs (127). miRNAs have been shown to participate in numerous aspects of immune regulation, including hematopoietic stem cell development, B and T-cell differentiation and maturation, neutrophil and macrophage proliferation, and cytokine production (128). Through their involvement in diverse biological processes and signaling pathways, miRNAs provide important insights into the molecular mechanisms responsible for the complex pathophysiology of MS. By modulating the expression and activity of a wide range of proteins, these regulatory molecules contribute to the diverse pathological processes associated with disease initiation and progression (126). Although many dysregulated miRNAs have been identified in patients with MS, the biological functions of only a few have been fully characterized. Among the dysregulated miRNAs, miR-96 has been reported to be upregulated in patients with MS. Its increased expression has been associated with the regulation of interleukin- and Wnt-mediated signaling pathways, suggesting an important role in the development and activation of both effector and regulatory T cells (44, 129). Another miRNA that has been extensively studied is miR-326, which is closely associated with T helper 17 (Th17) cells. miR-326 promotes Th17-cell differentiation by targeting ETS-1, a transcription factor that normally acts as a negative regulator of Th17 differentiation, thereby contributing to the pathogenesis of chronic autoimmune disorders such as MS (45). Petrocca et al. demonstrated that the expression levels of miR-19a, miR-19b, miR-25, and miR-106 are significantly elevated in Treg cells isolated from patients with MS compared with healthy controls. These miRNAs were shown to regulate the transforming growth factor-beta (TGF-β) signaling pathway through cyclin-dependent kinase inhibitor 1A (CDKN1A/p21) and BCL2-like protein 11 (BCL2L11), implicating them in impaired immune regulation during disease progression (127, 130). Expression of miR-21, miR-155, and miR-182 has also been detected in the cerebrospinal fluid of patients with MS. Their expression levels exhibit significant positive correlations with inflammatory mediators, including interleukin-1β (IL-1β), IL-6, TNF-α, and high-sensitivity C-reactive protein (hs-CRP), suggesting that these miRNAs actively contribute to the inflammatory processes characteristic of MS (46). Another study demonstrated that miR-467b directly targets eukaryotic translation initiation factor 4E (eIF4E), a molecule critically involved in Th17-cell differentiation. By modulating this pathway, miR-467b may delay disease progression, alter Th17-cell differentiation, and potentially prevent further neurological deterioration, highlighting its therapeutic potential in MS (47). Within active MS lesions, miR-155, miR-326, and miR-34a are consistently upregulated. These three miRNAs collectively target CD47, a membrane glycoprotein that regulates macrophage-mediated phagocytosis through interactions with signal regulatory protein alpha (SIRPα) expressed on myeloid cells (48). Additional miRNAs, including miR-92 and members of the let-7 family, have also been implicated in MS pathobiology. The target genes of miR-92 are primarily involved in cell-cycle regulation and intracellular signaling, whereas let-7 family members regulate stem cell differentiation, T-cell activation, and Toll-like receptor 7 (TLR7) signaling, all of which have been linked to neurodegenerative processes. These findings underscore the pivotal role of these miRNAs in the molecular mechanisms underlying MS (49). The miR-106b-25 cluster has likewise been reported to be significantly upregulated in PBMCs from patients with MS. Increased expression of this cluster has been proposed to disrupt TGF-β signaling, thereby promoting disease development by attenuating the suppressive phenotype of naïve T cells and impairing immune tolerance (131). Furthermore, miR-17-5p has been identified as a regulator of phosphatase and PTEN and phosphoinositide 3-kinase regulatory subunit 1 (PIK3R1), two genes involved in signaling pathways implicated in MS pathogenesis (50). The upregulation observed for several other miRNAs, including miR-145 and miR-155, has been reported to be significantly decreased in patients with relapsing-remitting multiple sclerosis (RRMS) compared with healthy controls. Both miRNAs are closely associated with the regulation of inflammatory pathways implicated in MS pathogenesis. Their reduced expression may contribute to disease development and progression. Further investigation of miR-145 and miR-155 may provide valuable insights into their precise biological functions and molecular mechanisms, potentially facilitating the development of novel therapeutic strategies for MS (126, 132).

### 3.4 Systemic lupus erythematosus

Systemic lupus erythematosus is a chronic, multi-systemic, complex autoimmune disease that can affect multiple organs and tissues and present with a variety of clinical manifestations. While the exact cause of the disease is not fully understood, numerous studies suggest that genetic predisposition, environmental and hormonal factors, and other factors may play a decisive role in its onset and progression. T cells can contribute to the development of SLE not only by directly interacting with other immune cells but also by releasing pro-inflammatory cytokines and acting directly on target tissues (133). miRNAs play diverse regulatory roles in innate immune responses and have been implicated in the pathogenesis of SLE through their involvement in immune cell dysfunction, abnormalities of resident tissue cells, and epigenetic dysregulation (134). Accumulating evidence indicates that miRNA expression is tightly regulated during immune cell development, differentiation, and effector function, as well as throughout the progression of immune-mediated disorders such as SLE. Although miRNAs regulate signaling pathways and autoimmune-related genes involved in lupus, their precise cellular and molecular mechanisms in the initiation and progression of SLE remain incompletely understood (49, 83). CD4^+^ T cells have consistently demonstrated increased expression of miR-21 and miR-148a in patients with SLE. Both miRNAs have been linked to DNA hypomethylation, a characteristic molecular abnormality associated with autoreactive immune responses in lupus. This epigenetic alteration is mediated through the suppression of DNA methyltransferase 1 (DNMT1) (51). miR-21 expression has also been associated with the abnormal phenotype of T lymphocytes in SLE, potentially through the inhibition of its target gene, PDCD4, a well-established tumor suppressor. Experimental silencing of miR-21 has been shown to reverse excessive T-cell proliferation, reduce IL-10 production, and decrease CD40 ligand (CD40L) expression (52). In addition, significantly increased expression of miR-29b (53) and miR-126 (54) has been reported in T cells isolated from patients with SLE. Both miRNAs contribute to reduced DNA methylation, thereby promoting aberrant activation of CD4^+^ T cells. In contrast, reduced expression of miR-181a has been observed in pediatric patients with SLE, suggesting its involvement in regulating B- and T-cell differentiation, maturation, and immune function (49, 55). Wang et al. demonstrated that serum and plasma levels of miR-146a are significantly lower in patients with SLE than in healthy controls and are negatively correlated with the Systemic Lupus Erythematosus Disease Activity Index (SLEDAI) (135). Similarly, serum exosomal miR-146a has also been reported to be downregulated in SLE. Exosomal miR-146a negatively regulates the senescence of bone marrow-derived mesenchymal stem cells by suppressing the TRAF6/NF-κB signaling pathway, highlighting its potential protective role in disease progression (56). Reduced expression of miR-125a has likewise been observed in patients with SLE. This miRNA regulates CCL5/RANTES expression by targeting Kruppel-like factor 13 (KLF13), a transcription factor expressed in activated T cells. Consequently, decreased miR-125a expression may contribute to enhanced inflammatory chemokine production and immune activation (57). miR-155 is another key regulator involved in the expansion and maturation of regulatory T cells, which are essential for maintaining immune tolerance and preventing autoimmunity. Upregulation of miR-155 has been detected in Treg cells from patients with SLE, where it suppresses CD62L expression, an adhesion molecule required for lymphocyte trafficking between the circulation and secondary lymphoid organs. This alteration contributes to an abnormal Treg phenotype and impaired immune regulation (58). Expression of miR-199a-3p has been reported in CD4^+^ T cells from patients with SLE. This miRNA exhibits a negative correlation with the expression of its target gene, signal-transducing adaptor molecule (STAM), a component of the Janus kinase/signal transducer and activator of transcription (JAK/STAT) signaling pathway, suggesting its involvement in dysregulated immune signaling (136). Overexpression of the miR-183 cluster (miR-183c) has also been implicated in SLE pathogenesis. Increased miR-183c suppresses the forkhead box protein O1 (FOXO1) gene, thereby promoting the differentiation of pro-inflammatory T helper cells and enhancing interferon-gamma (IFN-γ) production (59, 137). Furthermore, it has been shown that increased expression of miR-124-3p and miR-377-3p in PBMCs of SLE patients decreases the expression of early growth response protein-1 (EGR1), thereby increasing inflammatory responses (104). Several miRNAs, including miR-3148, let-7a, let-7c, and miR-125a, have also been implicated in regulating innate immune pathways associated with SLE by targeting genes involved in disease susceptibility (138). Among these, miR-3148 binds to the 3′UTR of toll-like receptor 7 (TLR7) mRNA in PBMCs, exhibiting an inverse correlation with TLR7 expression and suggesting a regulatory role in innate immune activation (139). Another study demonstrated that downregulation of miR-196a in SLE is associated with increased expression of the homeobox transcription factor Hox-C8, further supporting the contribution of miRNA-mediated transcriptional regulation to disease pathogenesis (60, 85). In pediatric patients, reduced expression has been observed; elevated plasma levels of miR-181a have been reported in adult patients with SLE and correlate with SLEDAI scores, indicating a potential association with disease activity (140). miR-181a has been shown to regulate inflammatory mediators, including IL-1β, IL-6, IL-8, and TNF-α, and to play a critical role in T- and B-cell differentiation and innate immune responses (141). A study reported that increased miR-1246 expression in B cells from patients with SLE suppresses early B-cell factor 1 (EBF1) expression, thereby impairing B-cell mobilization (61). Additionally, B-cell activating factor (BAFF), a crucial survival factor responsible for excessive B-cell activation in SLE, has been linked to increased miR-152-3p expression. Upregulated miR-152-3p targets and inhibits kruppel-like factor 5 (KLF5), thereby enhancing BAFF expression, promoting B-cell activation and mobilization, and ultimately contributing to excessive autoantibody production (62).

### 3.5 Inflammatory bowel disease

Inflammatory bowel disease is a chronic, multifactorial disorder characterized by a complex pathogenesis that has not yet been fully elucidated despite substantial advances in diagnostic approaches, pharmacological therapies, and surgical management (142). IBD is divided into two subtypes, each with its own unique clinical and pathological features: Ulcerative colitis (UC) and Crohn’s disease (CD). UC is characterized by persistent, superficial inflammation of the colon or rectal mucosa that can lead to erosion and ulceration. CD, on the other hand, affects any part of the GI tract from the mouth to the anus and is characterized by discontinuous transmural inflammation affecting all layers of the bowel wall. CD and UC mostly affect adolescents and cause abdominal pain, malabsorption, bloody diarrhea, weight loss, fatigue, and decreased quality of life. Long-term and uncontrolled inflammation also increases the likelihood of colorectal cancer (CRC) and raises the mortality rate to 10-15% (143). The incidence and prevalence of IBD have increased significantly, making it a global public health problem and necessitating the development of new treatment strategies. Recent studies have shown that miRNAs are differentially expressed in autoimmune diseases; in particular, miRNAs have been associated with critical inflammatory pathways involved in IBD pathogenesis (144). Increasing evidence indicates that miRNAs have pivotal roles in the initiation, progression, and maintenance of IBD by regulating the expression of both positive and negative components of immune signaling pathways and by modulating inflammatory and immune responses (142, 145). Acting as post-transcriptional regulators of gene expression, miRNAs contribute significantly to the pathogenesis of both UC and CD by influencing inflammatory signaling networks and intestinal immune homeostasis (146). Many studies have identified several miRNAs, including miR-122, miR-192, miR-155, miR-31, miR-146a, and miR-29, as key regulators involved in IBD pathophysiology (145, 146). In the first study demonstrating a direct association between miRNAs and IBD, Wu et al. reported that miRNAs regulate chemokine expression in colonic epithelial cells, thereby influencing intestinal inflammatory responses. In patients with active UC, miR-16, miR-21, miR-23a, miR-24, miR-29a, miR-126, miR-195, and let-7f were significantly upregulated compared with healthy controls. Conversely, expression levels of miR-188-5p, miR-215, miR-320a, and miR-346 were markedly reduced in inflamed colonic mucosa. The same study also demonstrated that miR-192 directly targets macrophage inflammatory peptide-2 (MIP-2) and is significantly downregulated in active UC (63). Subsequent investigations revealed that miR-192 suppresses nucleotide-binding oligomerization domain-containing protein 2 (NOD2) receptor activity in colonocytes, thereby limiting inflammatory activation and contributing to intestinal immune regulation (64, 146). Among the best-characterized inflammatory miRNAs in IBD, miR-21 is consistently overexpressed. miR-21 levels have been associated with enhanced T-cell activation in patients with UC in remission and with reduced expression of the tumor suppressor PDCD4 in CD3^+^ T cells, thereby promoting inflammatory progression and cellular proliferation (65). Notably, miR-21 is the only miRNA shown to correlate with both nitric oxide synthase 2 (NOS2) and CD68 expression in IBD. This association contributes to increased nitric oxide (NO) production and macrophage activation. Dysregulation of the NO signaling pathway further induces upregulation of heterochromatin protein 1γ (HP1γ), thereby promoting cellular senescence in adjacent intestinal epithelial cells (147). Yang et al. demonstrated that miR-21 overexpression disrupts the integrity of the intestinal epithelial barrier by targeting Ras homolog family member B (RhoB). This disruption results in decreased transepithelial electrical resistance, increased epithelial permeability, and enhanced epithelial apoptosis (66). In UC, elevated expression of miR-31 and miR-155 has been shown to regulate increased IL-13 activity by downregulating interleukin-13 receptor alpha 1 (IL13Rα1), the primary IL-13 receptor subunit (67). Furthermore, miR-31 is significantly upregulated in colonic tissues from patients with both UC and CD, and it directly targets interleukin-25 (IL-25). Consequently, the miR-31/IL-25 signaling axis modulates Th1- and Th17-mediated inflammatory responses in experimental models of colitis (148). Another study demonstrated that miR-155 directly targets E-cadherin, thereby reducing mucosal integrity in UC and potentially increasing the long-term risk of colorectal cancer progression (149). In CD, mutations in NOD2 have been associated with reduced miR-29 expression, leading to enhanced inflammatory responses and worsening colitis (68). Moreover, downregulation of miR-29b promotes intestinal fibrosis by increasing the expression of collagen type I and type III transcripts and the collagen type III protein, thereby contributing to fibrotic stricture formation in affected intestinal segments (150). Occludin and claudins are essential tight junction proteins that maintain epithelial barrier integrity against luminal bacterial antigens (142). miR-122 has been shown to directly target occludin mRNA, thereby increasing intestinal tight junction permeability through multiple regulatory mechanisms (69, 142). In addition, downregulation of the miR-200 family has been linked to dysregulated epithelial-to-mesenchymal transition (EMT) in CD through the loss of E-cadherin expression (151). Reduced expression of miR-141 has been consistently observed in colonic tissues from patients with both UC (70) and CD (71). This miRNA directly targets C-X-C motif chemokine ligand 5 (CXCL5) and CXCL12. Decreased miR-141 expression permits increased CXCL5 and CXCL12 activity, facilitating leukocyte recruitment and amplifying intestinal inflammatory responses. These findings suggest that miR-141 plays an important regulatory role in the pathogenesis of both UC and CD and may represent a promising therapeutic target (152). Another important inflammatory regulator, miR-146a, modulates intestinal inflammation through inducible nitric oxide synthase (iNOS)/NO-dependent regulation of Sonic Hedgehog (SHH) signaling. During intestinal inflammation, activation of the SHH pathway promotes the expression of several pro-inflammatory mediators, including IL-12, IL-6, TNF-α, and CCL5, thereby contributing to disease progression (153). Finally, Bian et al. demonstrated that miR-150 expression is significantly elevated in the inflamed colonic mucosa of patients with UC compared with healthy controls. miR-150 expression was inversely correlated with its target gene, the proto-oncogene c-Myb, suggesting that dysregulation of the miR-150/c-Myb axis may contribute to the pathogenesis of ulcerative colitis (72).

### 3.6 Atopic dermatitis

Atopic dermatitis is a common chronic inflammatory skin disease characterized by impaired skin barrier function and altered immune responses (154). This disease, also known as eczema, presents with severe itching (pruritus), dry skin, and recurrent eczematous lesions (155). The prevalence of AD is increasing, particularly in industrialized countries, affecting 15-30% of children and 2-10% of adults globally. AD is a chronic disease characterized by periods of exacerbation, and approximately 50-80% of patients also have other atopic disorders (154). The mechanism of atopic dermatitis development is not yet fully understood. However, it is known to be associated with disturbances in the epidermal barrier (including filaggrin structure and function, tight junctions, and lipid content), dysregulation of the immune system, and disorders originating from the neurovegetative system (155). Recent findings indicate that miRNAs play a significant role in the pathogenesis of AD. Dysregulation of miRNAs has been implicated in the pathogenesis of AD through its involvement in impaired skin barrier function, aberrant cytokine signaling, NF-κB-mediated inflammatory responses, and the regulation of Th17, Th1, Th2, and regulatory T cells, as well as platelet activity. Nevertheless, the precise biological functions of many AD-associated miRNAs remain unclear. Current evidence indicates that many miRNAs, including miR-223, miR-10a-5p, miR-29b, miR-146a-5p, miR-451a, miR-124, miR-143, miR-151a, miR-24, miR-191, miR-155, miR-1294, and miR-335, play significant roles in the development and progression of AD (155, 156). Among these, miR-223 negatively regulates Treg cell differentiation and immunosuppressive function, thereby contributing to immune dysregulation in AD (155). In contrast, miR-10a-5p influences keratinocyte proliferation, cell adhesion, and cytokine-mediated signaling pathways, suggesting an important role in maintaining epidermal homeostasis (157). The modulation of miR-29b expression has been proposed as a potential therapeutic strategy for AD (158). Both miR-146a-5p (159) and miR-124 (73) have emerged as key regulators of AD pathogenesis by modulating the NF-κB signaling pathway, thereby influencing immune responses. miR-143 has been identified as a critical regulator of Th2-mediated inflammation through its interaction with the IL-13 receptor alpha 1 (IL-13Rα1). IL-13, predominantly secreted by activated Th2 lymphocytes and mast cells, is widely recognized as a central cytokine driving allergic inflammation in AD (155). Experimental evidence suggests that miR-143 inhibits IL-13 signaling by targeting IL-13Rα1 in keratinocytes, thereby attenuating inflammatory responses and promoting the restoration of epidermal barrier integrity (74, 155). Increased expression of miR-151a has been shown to suppress interleukin-12 receptor beta 2 (IL12RB2), thereby reducing the production of Th1-associated cytokines. The consequent inhibition of Th1 responses may disrupt the Th1/Th2 immune balance and favor Th2-dominant inflammation (75). Chen et al. reported significantly higher miR-151a expression in peripheral blood cells from patients with AD than in those from healthy controls (75). Additional studies have demonstrated that miR-24 and miR-191 are also involved in AD pathogenesis. Serum levels of both miRNAs were positively correlated with thymus and activation-regulated chemokine (TARC/CCL17), a major chemoattractant that recruits Th2 cells, suggesting their potential utility as biomarkers of disease activity (160). The contribution of miR-155 to AD is multifaceted. It regulates tight junction formation in keratinocytes while simultaneously promoting Th17 cell differentiation (156, 161). Moreover, miR-155 directly targets cytotoxic T-lymphocyte-associated protein 4 (CTLA-4), an essential negative regulator of T-cell activation, thereby influencing immune cell activation and differentiation, particularly in T lymphocytes and dendritic cells (76). Interestingly, inhibition of miR-155 has been associated with increased GATA3 expression, the master transcription factor governing Th2 cell differentiation (162). Other studies have shown that miR-155 may suppress Th2 differentiation by inhibiting the transcription factor c-Maf (163). Despite these seemingly divergent findings, elevated miR-155 expression has consistently been associated with enhanced production of Th2 cytokines, including IL-5 and IL-13, underscoring its complex immunomodulatory role in AD (155). Yan et al. demonstrated that miR-1294 exerts anti-inflammatory effects by inhibiting the NF-κB signaling pathway through a STAT3-dependent mechanism while simultaneously suppressing reactive oxygen species (ROS)-mediated cellular responses (164). Additionally, miR-335 has been shown to directly inhibit the transcription factor SOX6 in the skin of patients with AD, thereby promoting keratinocyte differentiation and contributing to the maintenance of epidermal barrier function (77).

## 4 miRNAs as Biomarkers

miRNAs play a pivotal role in disease pathogenesis by regulating the expression of proteins involved in multiple molecular and cellular processes. Furthermore, an increasing number of studies suggest that alterations in miRNA expression serve as valuable biomarkers for detecting disease before clinical symptoms and for predicting disease severity, progression, and therapeutic response (79, 84). miRNAs are readily detectable in a wide range of biological fluids, including serum, plasma, saliva, tears, urine, amniotic fluid, and cerebrospinal fluid (83). More importantly, these molecules exhibit extraordinary stability, remaining resistant to enzymatic degradation, repeated freeze-thaw cycles, and extreme pH conditions (165). Their high stability, together with distinct expression profiles across different body fluids, makes miRNAs particularly attractive candidates for non-invasive diagnostic biomarkers (83).

Accumulating evidence indicates that miRNAs have considerable potential as both diagnostic and prognostic biomarkers for immune-mediated diseases. Some miRNAs identified as potential biomarkers for immune system-related diseases are shown in **Table 2**. In addition, they are expected to play a significant role in the development of future biologically targeted therapeutic strategies (166). Nevertheless, despite their promising clinical applications, comprehensive investigations are still required to validate their diagnostic accuracy, clinical utility, and therapeutic relevance before they can be routinely implemented in clinical practice. The potential of miRNAs as biomarkers is limited by several key challenges in clinical practice. Specifically, the ability of a single miRNA to modulate hundreds of target transcripts can pose challenges for diagnostic specificity. Furthermore, inter-study variations stemming from methodological differences and reproducibility issues are major obstacles limiting the routine clinical use of these molecules. Therefore, comprehensive studies are needed to validate diagnostic accuracy and clinical benefit before miRNA-based approaches can be integrated into precision medicine protocols.

**Table 2:** miRNAs are potential biomarkers for immune-related diseases.

miRNA	Biological sample	Expression	Disease	Diagnostic value	Ref
miR-132	Plasma	Up	RA	AUC=0.90	(89)
miR-24 miR-26a miR-125a-5p	Plasma	Up	RA	miR-125a-5p AUC = 0.83 miR-24 AUC = 0.80 miR-26a AUC = 0.80	(90)
miR-210 miR-155	Serum	miR-210(Down) miR-155 (Up)	RA		(91)
miR-223	PBMCs	Up	PS		(92)
miR-19a miR-29a	Serum	Down	PS		(93)
miR-369-3p	Serum Skin	Up	PS		(94)
miR-223 miR-143	PBMCs	Up	PS	miR-223 AUC = 0.80 miR-143 AUC = 0.75	(95)
miR-145	PBMCs	Up	MS	miR-145 AUC = 0.785	(96)
miR-219	CSF	Down	MS		(97)
miR-15b miR-223	Serum	Down	MS	miR-15b AUC: 0.75 miR-223 AUC: 0.80	(98)
miR-203 miR-365 miR-21 miR-520c-3p miR-191 miR-328 miR-140 miR-126 miR-199a-3p miR-143 miR-19a	CSF		MS	miR-150 AUC = 0.68 miR -645 AUC = 0.66 miR-30a-5p AUC = 0.65 miR-365 AUC = 0.64 miR-199-3pAUC=0.64 miR-106a AUC = 0.64 miR-328 AUC = 0.63 miR-21 AUC = 0.62 miR-191 AUC = 0.61 miR-146a AUC = 0.60	(99)
miR-326	Blood	Up	MS	AUC=1	(100)
miR-181c miR-633 miR-922	CSF		MS	miR-181c AUC = 0.73 miR-633 AUC = 0.82 miR-922 AUC = 0.74	(101)
miR-150	CSF	Up	MS	AUC=0.744	(102)
miR-181a miR-223	Serum	miR-181a (Up) miR-223(Down)	SLE	miR-181a AUC = 0.827 miR-223 AUC = 0.848	(103)
miR-124-3p miR-377-3p	PBMCs Serum		SLE	miR-124-3p AUC = 0.714 miR-377-3p AUC = 0.705	(104)
miR-371b-5p miR-5100	Serum	Up	SLE	miR-371b-5pAUC= 0.806 miR-5100 AUC = 0.810	(105)
miR-183-5p	PBMCs	Up	SLE	AUC= 0.703	(106)
miR-146a miR-155	Serum	Down	SLE		(107)
miR-31	Tissue	Up	IBD	AUC=0.75	(108)
miR-146b-5p	Serum	Up	IBD	AUC 0.869	(109)
miR-16	Plasma	Down	IBD	AUC=0.65	(110)
miR-24	Rectal Tissue Blood	Up	IBD	AUC= 0.83	(111)
miR-223	Plasma	Up	AD	AUC=0.837	(112)
miR-151a	Skin Tissue Peripheral Blood	Up	AD	AUC=0.8453	(75)
miR-451a	PBMCs	Up	AD	AUC=0.838	(113)
miR-194-5p	Plasma	Down	AD	AUC=0.784	(114)

RA, rheumatoid arthritis; PS, psoriasis; MS, multiple sclerosis; SLE, systemic lupus erythematosus; IBD, Inflammatory bowel disease; AD, atopic dermatitis AUC, area under the curve; PBMCs, peripheral blood mononuclear cells; CSF, cerebrospinal fluid.

### 4.1 miRNAs as biomarkers in RA

The clinical potential of miRNAs as early biomarkers is of considerable interest because they may detect disease-related molecular alterations before clinical symptoms. RA, timely diagnosis, and prompt initiation of treatment are essential to prevent or minimize irreversible joint destruction and disease progression. Therefore, identifying disease-specific miRNA expression profiles may facilitate the early diagnosis of RA and improve treatment (84). Murata et al. reported that miR-132 has the potential as a clinical biomarker for distinguishing patients with RA from those with osteoarthritis (OA). Receiver operating characteristic (ROC) curve analysis demonstrated that a plasma miR-132 threshold of 67.8 pmol/L discriminated RA patients with a sensitivity of 83.8%, a specificity of 80.7%, and an area under the curve (AUC) of 0.90, indicating strong diagnostic potential (89). Another study reported that circulating plasma miR-24, miR-26a, and miR-125a-5p may serve as highly specific diagnostic biomarkers for RA. Among these candidates, miR-125a-5p exhibited the highest diagnostic accuracy (AUC = 0.83), miR-24 (AUC = 0.80) and miR-26a (AUC = 0.80). Furthermore, multivariable logistic regression analysis identified miR-24, miR-30a-5p, and miR-125a-5p as key contributors to an RA diagnosis. Based on these findings, a plasma miRNA-based predictive algorithm (ePRAM) that integrates the expression levels of miR-24, miR-30a-5p, and miR-125a-5p was developed, thereby improving diagnostic accuracy with an AUC of 0.89 (90). Abdul-Maksoud et al. demonstrated that serum levels of miR-210 and miR-155 serve as independent diagnostic biomarkers for RA. Their findings further indicated that these miRNAs correlate with disease activity (91). While the pathogenesis of RA remains unclear, further studies on the roles of miRNAs will provide new insights into the complex gene regulatory network. Such advances could help identify novel diagnostic biomarkers and support the development of more accurate and effective strategies for the early diagnosis and treatment of RA.

### 4.2 miRNAs as biomarkers for Psoriasis

Many studies investigating psoriasis have consistently demonstrated aberrant miRNA expression profiles in affected individuals compared with healthy controls. Although substantial progress has been made in identifying miRNAs involved in the pathogenesis of psoriasis, further research is required to establish their clinical utility as non-invasive molecular biomarkers for disease diagnosis, prognosis, and monitoring of therapeutic response (117). Clinical investigations have revealed significant associations between circulating miRNA levels and Psoriasis Area and Severity Index (PASI) scores, highlighting their potential prognostic value. For example, circulating concentrations of miR-223 and miR-146a have been shown to correlate positively with PASI scores, suggesting that these miRNAs reflect the underlying inflammatory burden associated with disease severity (92). Oyama et al. identified serum miR-19a and miR-29a as potential biomarkers for psoriasis, demonstrating that miR-19a, in particular, has strong diagnostic value. Increased serum miR-19a expression was found to reflect activation of the TNF signaling pathway, emphasizing its relevance to disease pathophysiology (93). Another study reported that miR-369-3p expression in both serum and skin tissue samples from patients with psoriasis may serve as a valuable biomarker for assessing disease prognosis (94). Furthermore, expression levels of miR-143 and miR-223 were significantly elevated in PBMCs of patients with psoriasis. They showed positive correlations with PASI scores, indicating a close association with disease severity. ROC analyses demonstrated that both miR-223 (AUC = 0.80) and miR-143 (AUC = 0.75) possess promising diagnostic performance for distinguishing patients with psoriasis from healthy individuals. These findings support the growing evidence that circulating miRNAs are promising non-invasive biomarkers for the diagnosis, prognosis assessment, and treatment of psoriasis (95).

### 4.3 miRNAs as biomarkers for MS

Despite extensive research on MS and the growing understanding of its diverse pathological aspects, identifying reliable biomarkers that facilitate diagnosis, prognosis, and monitoring of therapeutic response remains a significant challenge (128). miRNAs have potential roles as both diagnostic and prognostic biomarkers as well as therapeutic targets in MS. Their differential expression in patients compared with healthy individuals, along with their detectability in easily accessible biological fluids such as blood and cerebrospinal fluid (CSF), further underscores their relevance in the study of this complex autoimmune disease (126). Søndergaard et al. reported that miR-145 (AUC = 0.785) exhibited the highest plasma expression levels in patients with MS and proposed its potential utility as a screening biomarker for the disease (96). In peripheral blood, miR-145 levels showed diagnostic performance, with a specificity of 89.5% and a sensitivity of 90.0% in distinguishing MS patients from healthy controls (167). Bruinsma et al. identified reduced miR-219 levels in the CSF of MS patients, suggesting its potential as a diagnostic biomarker (97). Serum miRNAs in MS were further investigated by Fenoglio et al. in two independent MS cohorts. Their findings revealed significantly lower levels of miR-15b (AUC = 0.75, 95% CI: 0.60–0.90) and miR-223 (AUC = 0.80, 95% CI: 0.66–0.93) in patients with MS. ROC curve analysis demonstrated that miR-223 achieved 80% diagnostic accuracy for distinguishing primary progressive MS from healthy controls. In comparison, miR-15b showed 75% diagnostic accuracy (98). In another study, Quintana et al. reported that several miRNAs were differentially expressed in MS patients: miR-203, miR-365, miR-21, miR-520c-3p, miR-191, miR-328, and miR-30a-5p were upregulated, whereas miR-140, miR-126, miR-199a-3p, miR-143, and miR-19a were downregulated. Among the evaluated candidates, miRNA-150 demonstrated the highest discriminatory power for MS diagnosis (AUC = 0.68), followed by miRNA-645 (AUC = 0.66), miRNA-30a-5p (AUC = 0.65), miRNA-365 (AUC = 0.64), miRNA-199-3p (AUC = 0.64), miRNA-106a (AUC = 0.64), miRNA-328 (AUC = 0.63), miRNA-21 (AUC = 0.62), miRNA-191 (AUC = 0.61), and miRNA-146a (AUC = 0.60) (99). In addition to their diagnostic utility, certain miRNAs may also reflect different stages and subtypes of MS. These molecules have been proposed as biomarkers capable of indicating the extent of neurodegenerative damage, the initiation of remyelination processes, and the activity of immune responses within the central nervous system (CNS), thereby assisting in disease staging and subtype differentiation (168). miR-326 (AUC = 1) in peripheral blood may serve as a biomarker capable of distinguishing between relapsing and remitting phases of MS (100).

Ebrahimkhani et al. identified a panel of nine miRNAs (miR-15b-5p, miR-23a-3p, miR-30b-5p, miR-223-3p, miR-374a-5p, miR-342-3p, miR-432-5p, miR-433-3p, and miR-485-3p) capable of differentiating RRMS from progressive MS. This represented one of the earliest demonstrations that miRNAs may serve not only as diagnostic biomarkers but also as robust tools for accurately predicting MS subtypes (169). In RRMS patients, miR-26a expression was significantly increased in PBMCs, reflecting its potential involvement in MS pathogenesis and disease progression (170). miRNA profiling in CSF samples from MS patients and healthy controls, identifying miR-181c, miR-633, and miR-922 as specifically expressed in MS. Moreover, miR-181c and miR-633 were able to differentiate secondary progressive MS (SPMS) from RRMS with sensitivities and specificities of 82% and 69%, respectively (101). Bergman et al. reported reduced levels of miR-150 in CSF, suggesting its potential role as an early diagnostic biomarker and a marker of inflammatory disease activity in MS (102). In addition, Gandhi et al. investigated a genome-wide analysis of serum miRNA expression at different stages of MS. They demonstrated that several miRNAs were differentially expressed across disease stages and that their expression levels correlated with the Expanded Disability Status Scale (EDSS). Specifically, miR-92a-1* and miR-145 differentiated RRMS from SPMS and healthy controls, while members of the let-7 family distinguished SPMS from RRMS and controls. Additionally, miR-454 was able to discriminate RRMS from SPMS. miR-145 has previously been validated as a diagnostic biomarker in MS studies. These findings highlight circulating miRNAs as accessible and informative biomarkers for disease monitoring, staging, and classification in MS (83, 171).

### 4.4 miRNAs as biomarkers for SLE

The multifactorial nature and complex pathogenesis of SLE present considerable challenges for its clinical classification and diagnosis (138). In routine clinical practice, the diagnosis of SLE remains difficult owing to the lack of highly sensitive and specific biomarkers (172). Furthermore, the unpredictable therapeutic responses observed among patients highlight the need for biomarkers to improve understanding of disease mechanisms and facilitate the identification of novel therapeutic targets (138). Increasing research efforts have focused on miRNAs as potential biomarkers for the diagnosis and prognosis of SLE (172). Evidence from the literature suggests that circulating miR-181a (AUC = 0.827) and miR-223 (AUC = 0.848) may serve as novel serum-based biomarkers for assessing disease progression and prognosis in patients with SLE (103). In another study, expression levels of miR-124-3p and miR-377-3p were significantly higher in both PBMCs and serum samples from patients with SLE compared with those from healthy controls (P < 0.05). ROC curve analysis demonstrated that miR-124-3p yielded an AUC: 0.714 (95% confidence interval [CI]: 0.610–0.820; sensitivity 70.0%; specificity 68.1%), whereas miR-377-3p achieved an AUC of 0.705 (95% CI: 0.600–0.809; sensitivity 72.0%; specificity 57.4%). These findings indicate that miR-124-3p and miR-377-3p have promising diagnostic potential for SLE. Moreover, a combined diagnostic model incorporating plasma miR-124-3p and miR-377-3p further improved diagnostic performance, yielding an AUC of 0.744 (95% CI: 0.640–0.850) (104). Zeng et al. reported that the expression levels of miR-371b-5p and miR-5100 in both CD4^+^ and CD8^+^ T cells can effectively distinguish patients with active SLE from those with inactive disease. Correlation analyses further demonstrated that the expression of both miRNAs was positively associated with SLEDAI scores. ROC analysis revealed miR-371b-5p (AUC: 0.806, 95% CI: 0.785–0.828) and miR-5100 (AUC: 0.810, 95% CI: 0.789–0.832) when differentiating patients with SLE from healthy controls, highlighting their potential utility as biomarkers of disease activity (105). Zhou et al. identified miR-183-5p as a promising biomarker for SLE. Increased miR-183-5p expression was positively correlated with both SLEDAI scores and anti-double-stranded DNA (anti-dsDNA) antibody levels, indicating its association with disease severity. To evaluate the diagnostic performance of miR-183-5p and miR-374b-3p, ROC curve analyses were performed. The AUC values were for 0.703 (95% CI: 0.574–0.833) for miR-183-5p and 0.681 (95% CI: 0.542–0.826) for miR-374b-3p. Notably, combining these two miRNAs substantially improved diagnostic accuracy, resulting in an AUC of 0.832 (95% CI: 0.727–0.937). These findings suggest that both miR-183-5p and miR-374b-3p have diagnostic value for SLE, and that their combined assessment offers superior diagnostic performance compared with either biomarker alone (106). The first evidence supporting miRNAs as non-invasive biomarkers for SLE was reported by Wang et al. through the evaluation of serum miR-146a and miR-155 levels. Their study demonstrated that both miR-146a and miR-155 were significantly reduced in patients with SLE compared with healthy controls. Furthermore, serum miR-146a levels showed an inverse correlation with proteinuria and SLEDAI scores, indicating a close association with disease activity (107). Serum levels of miR-200a, miR-200b, miR-200c, miR-429, miR-205, and miR-192 were significantly lower in patients with SLE than in healthy individuals. Importantly, the expression levels of these miRNAs were significantly associated with SLEDAI scores, suggesting that they serve as valuable indicators of disease activity (173). These findings support the growing evidence that serum miRNAs have potential as minimally invasive biomarkers for the diagnosis and prognostic evaluation of SLE.

### 4.5 miRNAs as biomarkers for IBD

lthough substantial progress has been made in elucidating the complex pathophysiology of IBD, the identification of highly specific and accurate non-invasive biomarkers for diagnosis and disease monitoring remains an unmet clinical need (174). Diagnostic challenges also persist in distinguishing UC from CD, particularly when inflammatory lesions are confined to the colon, as well as in differentiating IBD from irritable bowel syndrome (IBS) (146). Because miRNAs are remarkably stable in peripheral blood, saliva, and stool, they have emerged as promising non-invasive biomarkers with potential applications in the early diagnosis, prognostic assessment, and monitoring of disease remission in IBD (146). Lin et al. identify miRNAs that could be used to diagnose IBD and distinguish UC from CD. Their study demonstrated significant expression of miR-31, miR-146a, miR-206, and miR-424 in fresh-frozen intestinal tissue samples from patients with CD compared with healthy controls, and similar expression patterns were subsequently confirmed in patients with UC. Furthermore, miR-31 was consistently upregulated in formalin-fixed, paraffin-embedded colonic mucosal samples from patients with both UC and CD, demonstrating moderate diagnostic performance (AUC: 0.75) with 71% sensitivity and 67% specificity. Notably, miR-31 expression is independent of disease activity, suggesting that it serves as a stable biomarker for IBD and facilitates differentiation from other forms of colitis (108). Chen et al. reported that serum miR-146b-5p expression was approximately 2.87-fold higher in patients with regional ileitis and 2.72-fold higher in patients with IBD than in healthy controls. Serum miR-146b-5p levels were significantly associated with disease activity and demonstrated greater disease specificity than C-reactive protein (CRP), highlighting its potential clinical value (AUC: 0.869) (109). Several studies have further demonstrated that serum levels of miR-16, miR-21, and miR-223 are significantly elevated in patients with IBD compared with healthy individuals, with expression levels generally higher in CD than in UC (175). Paraskevi et al. identified circulating miRNA signatures that differentiate CD from UC. miR-16, miR-23a, miR-29a, miR-106a, miR-107, miR-126, miR-191, miR-199a-5p, miR-200c, miR-362-3p, and miR-532-3p were significantly overexpressed in the peripheral blood of patients with CD compared with healthy controls. In contrast, patients with UC exhibited markedly increased expression of miR-16, miR-21, miR-28-5p, miR-151-5p, miR-155, and miR-199a-5p (176). Comparative analyses between active and inactive CD identified six differentially expressed miRNAs: miR-188-5p, miR-877, miR-140-5p, miR-145, miR-18a, and miR-128 that may reflect disease activity (177). Jensen et al. showed the diagnostic potential of plasma miR-16, miR-106a, and miR-140-3p in CD. In an independent validation cohort of 102 patients with CD, plasma miR-16 expression was significantly reduced compared with that in individuals without CD (fold change = 0.83, *p* = 0.02). ROC analysis yielded an area AUC of 0.65, indicating diagnostic performance (110). In addition, miR-31 has been proposed as a marker of genetic susceptibility to CD (178). Radha et al. reported a diagnostic miRNA panel comprising 31 miRNAs that distinguished patients with UC from healthy individuals, achieving an overall diagnostic accuracy of 92.8%, specificity of 96.2%, and sensitivity of 89.5%, underscoring the potential of multi-marker approaches for clinical diagnosis (179). Expression of miR-24 has also been shown to differ significantly between UC and L2 Crohn’s disease (Crohn’s colitis) in rectal biopsy specimens, as determined by qRT-PCR. ROC analysis demonstrated that rectal miR-24 correctly classified 84.2% of patients, achieving a sensitivity of 83.3% and a specificity of 85.7%. These findings suggest that miR-24 is a valuable biomarker for distinguishing UC from colonic CD (111). A panel of differentially expressed miRNAs, including miR-19a, miR-21, miR-31, miR-101, miR-146a, and miR-375, was used in matched colonic biopsy samples obtained from patients with CD and seven patients with UC. Statistical analyses demonstrated that this miRNA signature possesses significant discriminatory power for differentiating CD from UC, further supporting the growing role of miRNA profiling as a promising diagnostic strategy for IBD (180).

### 4.6 miRNAs as biomarkers for AD

Altered miRNA expression profiles have been consistently identified in the skin and serum of patients with AD, suggesting their potential value as diagnostic and prognostic biomarkers (181). In patients with severe AD, plasma miR-223 expression levels were significantly higher than those observed in individuals with mild or moderate disease. The reported area under the ROC curve, AUC = 0.837 (95% confidence interval [CI]: 0.614–1.000), indicates good diagnostic performance. These findings suggest that miR-223 is associated with the mechanisms underlying disease exacerbation and may serve as a promising biomarker for identifying severe AD (112). miR-151a has been reported to exhibit altered expression patterns in patients with AD compared with healthy individuals. Detection of miR-151a in skin tissue or peripheral blood has been proposed as a complementary biomarker that may enhance the diagnostic accuracy of existing clinical criteria for AD (75). Distinct expression of miR-451a has also been observed across different blood compartments in patients with AD. Owing to its disease-specific expression profile, miR-451a (AUC = 0.838) has been proposed as a promising biomarker candidate for the early detection of AD (113). Furthermore, reduced plasma miR-194-5p expression has been reported in pediatric patients with AD. This finding suggests that miR-194-5p (AUC = 0.784) may serve as a valuable non-invasive biomarker for the early diagnosis of this inflammatory skin disorder, particularly in the pediatric population (114). Despite encouraging diagnostic performance reported in many studies, most miRNA biomarkers have not yet undergone large-scale multicenter validation. Differences in sample preparation, normalization strategies, detection platforms, and patient heterogeneity remain major obstacles preventing their routine clinical implementation.

## 5 miRNA as therapeutic target for immune-mediated diseases

miRNAs are increasingly recognized not only as valuable biomarkers for the early detection and prognostic assessment of diseases, but also as promising therapeutic targets for the treatment of immune-mediated disorders (182). Modulation of miRNA expression enables precise regulation of inflammatory responses, allowing either the suppression of pathological inflammation or the enhancement of anti-inflammatory pathways (183, 184). Consequently, the application of RNA interference technologies to manipulate miRNA profiles has attracted growing scientific interest (127). Current miRNA-based therapeutic strategies are generally categorized into two principal approaches: miRNA mimics and miRNA antagonists. miRNA mimics (AgomiRs) are designed to restore the function of miRNAs that become downregulated during disease progression, thereby compensating for the loss of their regulatory activity.

In contrast, AntagomiRs are used to inhibit the effects of aberrantly upregulated miRNAs and reduce their pathogenic influence (182). The therapeutic efficacy of miRNA-based interventions largely depends on the ability to deliver miRNA modulators to target tissues in a specific, efficient, and safe manner (145). The literature describes five major delivery platforms: viral vectors, exosomes, molecular conjugates, lipid-based carriers (including lipid nanoparticles and liposomes), and polymeric delivery systems (143, 185). Although numerous preclinical studies have demonstrated encouraging therapeutic efficacy, only a limited number of miRNA-based therapeutics have advanced into clinical trials. Major challenges include target specificity, off-target effects, immune activation, pharmacokinetics, and efficient tissue-specific delivery. Therefore, further optimization of delivery technologies such as lipid nanoparticles, engineered exosomes, viral vectors, and polymer-based systems is required before widespread clinical application becomes feasible. Given their roles in immune responses and their dysregulation in the pathogenesis of immune-mediated diseases, the number of studies investigating miRNA-targeted therapies continues to increase, highlighting their potential as a novel class of immunomodulatory treatments (138, 185). Current evidence from the literature suggests that several miRNAs may serve as promising therapeutic targets for RA. Experimental studies have demonstrated that modulation of specific miRNAs can influence key pathogenic mechanisms involved in disease progression. In an *in vitro* model, administration of miR-124a suppressed the proliferation of rheumatoid arthritis synovial fibroblasts (RASFs) by inducing cell-cycle arrest. Moreover, miR-124a reduced the expression of CDK2 and MCP-1, thereby limiting the chemotactic recruitment of inflammatory cells (186). In another experimental arthritis study, administration of miR-34a inhibitors resulted in a marked improvement in arthritic manifestations. These therapeutic effects were associated with reduced T-cell infiltration, decreased pro-inflammatory cytokine expression, and attenuation of bone destruction (28). Furthermore, lentiviral overexpression of miR-140-3p and miR-140-5p has been reported to promote apoptosis by targeting sirtuin 1 (SIRT1) and stromal cell-derived factor 1 (SDF-1/CXCL12), respectively, while concurrently suppressing cellular proliferation, migration, and inflammatory cytokine production (187). The ability of miRNAs to regulate multiple molecular pathways involved in inflammation, immune responses, and tissue destruction highlights their significant potential for the development of novel miRNA-based therapeutic strategies for rheumatoid arthritis. miRNA-targeted therapeutic approaches have emerged as a promising strategy for treating psoriasis, primarily because of their ability to restore epidermal homeostasis and modulate aberrant immune cell infiltration (188). In imiquimod (IMQ)-induced murine models of psoriasis, inhibition of miR-21 or miR-31 significantly reduced epidermal hyperplasia, inflammatory immune cell infiltration, and the expression of pro-inflammatory cytokines (184). Restoration of the diminished expression of miR-125b or miR-99a has been shown to re-establish keratinocyte differentiation by suppressing the STAT3 and NF-κB signaling pathways.

Furthermore, administration of therapeutic miRNA mimics has demonstrated beneficial effects on disease severity (189). Using locked nucleic acid (LNA)-modified anti-miR-21 oligonucleotides, Guinea-Viniegra et al. demonstrated that anti-miR-21 therapy represents a promising therapeutic strategy for psoriasis (190). Likewise, Xue et al. reported that topical administration of a miR-205-5p mimic to psoriatic skin lesions markedly alleviated psoriasis-like symptoms in mice, an effect associated with inhibition of MAPK and Wnt/β-catenin signaling pathways (191). Additional preclinical studies have further highlighted the therapeutic potential of miRNA modulation in psoriasis. Intravenous administration of a miR-340 mimic reduced disease severity by suppressing IL-17A expression (192). Similarly, intradermal delivery of synthetic miR-146 mimics effectively attenuated IL-17A-mediated cutaneous inflammation, inhibited neutrophil infiltration, and reduced epidermal thickening (32). Moreover, local administration of AgomiR-145-5p into skin tissue substantially diminished epidermal hyperplasia and significantly improved psoriasis-like dermatitis in murine models (193).

In MS, suppression of pathogenic Th17 cell differentiation is widely regarded as a fundamental therapeutic strategy for limiting disease progression. Among the miRNAs investigated, miR-30a has emerged as a key regulatory molecule. Its overexpression directly targets the messenger RNAs of the interleukin-21 receptor (IL-21R) and interferon regulatory factor 4 (IRF4), thereby inhibiting Th17 cell differentiation and reducing disease severity in experimental autoimmune encephalomyelitis (EAE), the most commonly used animal model of MS. Other miRNAs, including miR-146a, have likewise been identified as important modulators of inflammatory gene expression due to their capacity to regulate and suppress Th17 cell differentiation. By constraining the development of this pathogenic T-cell subset, miR-146a helps attenuate autoimmune inflammatory responses, highlighting its potential therapeutic relevance in MS (194). Moreover, Liu et al., using a murine EAE model, demonstrated that miR-15b-mediated downregulation of O-linked N-acetylglucosamine transferase (OGT) significantly suppressed Th17 cell differentiation. These findings suggest that targeting the miR-15b/OGT axis may interfere with the pathogenic mechanisms underlying MS and represent a promising therapeutic approach for modulating disease progression (195).

miRNA-focused approaches in the treatment of SLE aim to target specific molecular pathways implicated in the disease, potentially offering more precise and effective therapy while reducing the need for broad immunosuppression (138). In PBMCs obtained from patients with SLE, miR-101-3p is overexpressed and directly targets histone deacetylase 9 (HDAC9). Through this mechanism, miR-101-3p suppresses the differentiation of CD4^+^ T cells into the pathogenic Th17 lineage, highlighting its potential as a novel therapeutic target (196). Preclinical studies have also demonstrated encouraging therapeutic effects for several other miRNAs. In MRL/lpr mouse models, enhanced expression of miR-590-3p significantly ameliorated lupus nephritis and cutaneous lesions, suggesting a promising strategy for improving clinical manifestations of SLE (197). Similarly, miR-125a has been proposed as a therapeutic candidate for its ability to regulate the expression of inflammatory chemokines, thereby modulating immune-mediated inflammatory responses associated with the disease (57). In addition, studies using MRL/lpr lupus-prone mice have shown that treatment with a miR-7 antagomir effectively reversed both the immunological abnormalities and pathological manifestations of lupus. These therapeutic effects were mediated through modulation of the PI3K/Akt signaling pathway and interleukin-21 (IL-21) activity. Collectively, these findings underscore the pivotal role of miR-7 in the pathogenesis of SLE and support its potential as a promising target for miRNA-based therapeutic strategies (198).

Many studies in IBD have shown that miRNA-based interventions are effective in experimental models. Among the miRNAs investigated, miR-301a has been shown to promote Th17 cell differentiation through the direct regulation of Smad nuclear interacting protein 1 (SNIP1) and in 2, 4, 6-trinitrobenzene sulfonic acid (TNBS)-induced colitis models, rectal administration of a miR-301a inhibitor significantly reduced the expression of pro-inflammatory cytokines within inflamed colonic tissue, indicating its therapeutic potential in suppressing intestinal inflammation (199). Similarly, inhibition of miR-30c and miR-130a using anti-miR oligonucleotides attenuated intestinal inflammation in a murine ileal loop model (200). Additional studies employing IL-10 knockout mice and TNBS-induced colitis models further demonstrated the regulatory role of miR-141 in intestinal inflammation. Intracolonic administration of an anti-miR targeting miR-141 exacerbated disease severity, whereas delivery of a pre-miR mimic to enhance miR-141 expression significantly alleviated intestinal inflammation, highlighting the protective role of this miRNA (71). Another promising therapeutic candidate is miR-155, whose inhibition has been shown to modulate the JAK signaling pathway by targeting a key regulatory protein involved in this cascade (201). Consequently, treatment with a miR-155 antagomir produces effects comparable to those achieved with clinically approved JAK inhibitors, which are currently used to manage UC (202). Furthermore, inhibition of miR-122a, miR-7a-5p, miR-155, and miR-223 has been reported to enhance the expression of tight junction (TJ) proteins in experimental models of UC and CD. By strengthening intestinal barrier integrity, these interventions help preserve epithelial function and reduce intestinal permeability, thereby attenuating disease severity (69, 203–205).

Modulation of miRNA expression has emerged as a promising therapeutic strategy for managing AD, owing to its potential to regulate inflammatory responses and restore skin homeostasis precisely (206). Studies in mouse models have shown that treatment with anti-miR-155-5p inhibitors significantly alleviates atopic inflammation by reducing epidermal thickening and suppressing the production of Th2-related cytokines, including IL-4, IL-5, IL-9, and IL-13. These findings suggest that anti-miR-155-5p is a promising therapeutic candidate for AD (207). Additional investigations have highlighted the therapeutic potential of miRNA mimics. Administration of AgomiR-10a-5p, which targets hyaluronan synthase 3 (HAS3), has been shown to reduce keratinocyte proliferation together with the expression of the pro-inflammatory mediators IL-8 and CCL5. Likewise, overexpression of AgomiR-124 in keratinocytes suppresses the NF-κB p65 subunit, thereby decreasing IL-8, CCL5, and CCL8 levels and attenuating inflammatory signaling (184). Furthermore, other miRNA-based approaches have demonstrated anti-inflammatory effects in experimental models of AD. Administration of a miR-143 mimic effectively suppressed IL-13-mediated inflammatory responses, whereas a miR-146a mimic directly targeted TRAF6 and IRAK1, leading to inhibition of Toll-like receptor 2 (TLR2)-mediated production of IL-8, CCL20, and TNF-α (182). Additionally, inhibition of IL-32 by miR-205 has been shown to deactivate the NF-κB signaling pathway in mouse models of AD, thereby reducing inflammatory responses. These findings further support the therapeutic potential of miR-205 and underscore the broader potential of miRNA-targeted interventions as innovative treatment strategies for AD (208).

Overall, accumulating experimental evidence indicates that miRNA-based therapies can modulate multiple inflammatory and immunological pathways implicated in immune-mediated diseases. Investigating the therapeutic potential of miRNAs can lead to “personalized” treatments while also reducing the risk of side effects. Supporting the transfer of miRNA-based therapeutic approaches to clinical practice is crucial. Despite promising results from miRNA-based therapies in preclinical models, critical technical and biological barriers remain to be overcome to translate these approaches into clinical practice. Key challenges limiting clinical use include target specificity, off-target effects, potential immune activation, toxicity, and pharmacokinetic limitations. Furthermore, maintaining the stability of therapeutic components within the body and ensuring their efficient delivery and uptake by target tissues is one of the biggest methodological obstacles. To improve clinical success and optimize safety, the development of carrier technologies, including lipid nanoparticles, engineered exosomes, viral vectors, and polymer-based systems, is being emphasized. In addition, increasing target specificity through the rational design of miRNA mimics and inhibitors will enable the development of more precise and “personalized” treatment strategies by minimizing unintended molecular interactions. Addressing these challenges will enable miRNA therapeutics to reach their full potential in immune-mediated diseases.

## 6 Conclusion and future perspective

miRNAs, recognized as key regulators of gene expression, have been shown to participate in a wide range of biological processes, including immune system homeostasis and the pathogenesis of immune-related disorders. These small non-coding RNAs exert their regulatory effects through multiple molecular mechanisms, most notably by modulating mRNA stability and translation, and by influencing several critical signaling pathways, including MAPK, JAK/STAT, and Wnt signaling cascades. Disease-specific miRNA expression signatures in immune-mediated disorders are essential for elucidating the molecular mechanisms by which dysregulated miRNAs contribute to disease pathogenesis. Such investigations may improve our understanding of the biological basis of miRNA dysregulation and facilitate the identification of novel miRNA-based biomarkers for disease diagnosis, prognosis, prevention, and therapeutic intervention. miRNAs are particularly promising biomarker candidates because they can improve the sensitivity and specificity of disease diagnosis and enable more precise monitoring of therapeutic responses. Moreover, although several technical and biological challenges remain before widespread clinical implementation, miRNA-based applications have the potential to facilitate the development of safer, more targeted, and personalized therapeutic strategies for immune-mediated diseases. In conclusion, despite substantial progress in elucidating the biological functions of miRNAs, the molecular mechanisms by which miRNA regulation or dysregulation contributes to the initiation, progression, or prevention of immune-mediated diseases remain only partially understood. Future research should focus on integrating multi-omics technologies, including transcriptomics, proteomics, metabolomics, single-cell sequencing, and spatial transcriptomics, with artificial intelligence assisted network analysis to identify robust disease specific miRNA signatures. Such integrative approaches are expected to accelerate the development of personalized diagnostic algorithms and precision medicine strategies for immune mediated diseases. Consequently, further experimental and clinical studies are required to clarify these complex regulatory networks and to accelerate the translation of miRNA-based biomarkers and therapeutic approaches into clinical practice.
